# The ABA-responsive transcription factor *SmMYB32* positively modulates tanshinone biosynthesis in *Salvia miltiorrhiza*

**DOI:** 10.1093/hr/uhag186

**Published:** 2026-11-12

**Authors:** Ying Xu, Ruiyan Zhu, Qinzhe Lin, Chenge Yu, Siwei Zhang, Jiaochen Qi, Jie Sun, Tingyao Liu, Qikai Huang, Lili Shao, Miaomiao Sheng, Yao Wang, Min Shi, Guoyin Kai

**Affiliations:** Laboratory of Medicinal Plant Biotechnology, School of Pharmaceutical Sciences, Zhejiang Chinese Medical University, Hangzhou 310053, China; Laboratory of Medicinal Plant Biotechnology, School of Pharmaceutical Sciences, Zhejiang Chinese Medical University, Hangzhou 310053, China; Laboratory of Medicinal Plant Biotechnology, School of Pharmaceutical Sciences, Zhejiang Chinese Medical University, Hangzhou 310053, China; Laboratory of Medicinal Plant Biotechnology, School of Pharmaceutical Sciences, Zhejiang Chinese Medical University, Hangzhou 310053, China; Laboratory of Medicinal Plant Biotechnology, School of Pharmaceutical Sciences, Zhejiang Chinese Medical University, Hangzhou 310053, China; Laboratory of Medicinal Plant Biotechnology, School of Pharmaceutical Sciences, Zhejiang Chinese Medical University, Hangzhou 310053, China; Laboratory of Medicinal Plant Biotechnology, School of Pharmaceutical Sciences, Zhejiang Chinese Medical University, Hangzhou 310053, China; Laboratory of Medicinal Plant Biotechnology, School of Pharmaceutical Sciences, Zhejiang Chinese Medical University, Hangzhou 310053, China; Laboratory of Medicinal Plant Biotechnology, School of Pharmaceutical Sciences, Zhejiang Chinese Medical University, Hangzhou 310053, China; Laboratory of Medicinal Plant Biotechnology, School of Pharmaceutical Sciences, Zhejiang Chinese Medical University, Hangzhou 310053, China; Laboratory of Medicinal Plant Biotechnology, School of Pharmaceutical Sciences, Zhejiang Chinese Medical University, Hangzhou 310053, China; Laboratory of Medicinal Plant Biotechnology, School of Pharmaceutical Sciences, Zhejiang Chinese Medical University, Hangzhou 310053, China; Laboratory of Medicinal Plant Biotechnology, School of Pharmaceutical Sciences, Zhejiang Chinese Medical University, Hangzhou 310053, China; Laboratory of Medicinal Plant Biotechnology, School of Pharmaceutical Sciences, Zhejiang Chinese Medical University, Hangzhou 310053, China

## Abstract

Tanshinones are natural products primarily biosynthesized in the root of *Salvia miltiorrhiza*. Their accumulation can be promoted by abscisic acid (ABA) treatment; however, the underlying regulatory mechanisms remain largely unclear. Here, an ABA-responsive R2R3-MYB transcription factor *SmMYB32* was identified from the ABA-induced transcriptome. Overexpression of *SmMYB32* significantly increased the tanshinone content in transgenic *S. miltiorrhiza* hairy roots by upregulating the expression of tanshinone-biosynthesis genes *SmCYP76AH3* and *SmCYP71D411*, whereas RNA interference-mediated silencing of *SmMYB32* produced the opposite effect. Further analysis using yeast one-hybrid, dual-luciferase reporter, and electrophoretic mobility shift assays demonstrated that SmMYB32 directly activates the transcription of *SmCYP76AH3* and *SmCYP71D411*. Furthermore, SmMYB32 physically interacts with the ABA receptor SmPYL8, and this interaction inhibits the transcriptional activity of *SmCYP76AH3* and *SmCYP71D411*. These findings uncover a novel regulatory mechanism underlying tanshinone biosynthesis and provide new insights into metabolic engineering in *S. miltiorrhiza*.

## Introduction

*Salvia miltiorrhiza* Bunge is a renowned traditional Chinese medicine extensively used in treatment of cardiovascular and cerebrovascular diseases [1, 2]. Tanshinones constitute a group of bioactive compounds in *S. miltiorrhiza* that predominantly exhibit a diterpenoid quinone structure, characterized by distinctive orange–yellow to orange–red hues [3]. More than 40 types of tanshinones have been identified, with major components including tanshinone I (TI), tanshinone IIA (TIIA), cryptotanshinone (CT), and dihydrotanshinone I (DT). These compounds exhibit various pharmacological activities such as antioxidant, antitumor and antiatherosclerotic [4–6]. Tanshinone biosynthesis and accumulation are tissue-specific and developmentally regulated. Specifically, tanshinones predominantly accumulate in the roots, with biosynthesis initiating as early as 25 days after germination [7].

The biosynthetic pathways of tanshinones have been extensively studied. Isopentenyl pyrophosphate (IPP) and dimethylallyl pyrophosphate (DMAPP) are common precursors for tanshinone biosynthesis. These originate from the mevalonate (MVA) pathway in the cytoplasm and the 2-C-methyl-D-erythritol 4-phosphate (MEP) pathway in plastids, respectively. IPP and DMAPP, in a ratio of 3:1, are condensed by geranylgeranyl diphosphate synthase (GGPPS) to yield geranylgeranyl diphosphate (GGPP), which serves as the direct precursor of tanshinones [8, 9]. Subsequently, miltiradiene is generated from GGPP through the catalytic action of copalyl-diphosphate synthase (CPS) and ent-kaurene synthase-like (KSL), and followed by a series of reactions catalyzed by specific cytochrome P450s enzymes including CYP76AH1, CYP76AH3, CYP76AK1, CYP71D373, CYP71D375, and CYP71D411, ultimately leading to the production of tanshinones [9, 10].

In addition to the parent plant, hairy roots also serve as an effective system for tanshinone production [11–13]. Due to the relatively low content of tanshinones in *S. miltiorrhiza*, various approaches have been applied to elevate the production. Genetic engineering of key tanshinone-biosynthetic genes involved in MVA and/or MEP pathways has been proved to be a feasible method [8, 14]. For example, coexpression of *SmGGPPS* and *SmDXS2* achieved higher tanshinone content than control [14]. The application of elicitors has also been shown to effectively enhance tanshinone production and combining genetic engineering with elicitation is also effective for boosting tanshinone production [15]. Abscisic acid (ABA) treatment has been reported to promote the accumulation of tanshinones [16]. In plants, the ABA signaling cascade is initiated when the ABA is perceived by intracellular receptors of the PYR/PYL/RCAR family, and then ABA-bound receptors inhibit the PP2C phosphatases, thereby relieving their suppression of sucrose nonfermenting 1-related protein kinases 2 (SnRK2s), allowing them to phosphorylate a range of targets including AREB/ABFs [17, 18]. However, the specific regulatory mechanism by which ABA in tanshinone biosynthesis remains unclear.

Transcription factors (TFs) play diverse roles in plants by modulating the expression of target genes. MYB TFs represent one of the largest TF families in plants, featuring varying numbers of highly conserved MYB repeat domains at their N-terminus that mediate DNA binding. These domains typically consist of one to four tandem, incompletely repeated amino acid sequences (R1, R2, R3, and R4) [19]. Based on the types and numbers of MYB repeats, the MYB TF family can be classified into four subfamilies: R1R2R3R1/2-MYBs (4R-MYBs), R1R2R3-MYBs (3R-MYBs), R2R3-MYBs, and MYBs (1R-MYB or MYB-related) [20]. Among these, R2R3-MYB proteins are the most prevalent in plants and play crucial roles in regulating various biological processes, such as secondary metabolism [21]. For instance, CmMYB9a enhanced floral coloration by activating *CmCHS*, *CmDFR*, and *CmFNS* in chrysanthemum [22]. Overexpression (OE) of *MdMYB9* or *MdMYB11* promoted both anthocyanin and proanthocyanidin accumulation in apple [23], whereas PtrMYB57 negatively regulated these pathways in poplar [24]. In *S. miltiorrhiza*, 110 R2R3-MYBs have been identified and classified into 37 subgroups [25]. Several R2R3-MYBs have been identified as regulators of tanshinone biosynthesis. For instance, SmMYB98 functioned as an activator in tanshinone biosynthesis [26], SmMYB53 activated *SmCYP71D375* to positively regulate tanshinone accumulation [27], and SmMYB36 enhanced tanshinone biosynthesis by activating *SmDXS2*, *SmGGPPS1*, and *SmCPS1* [28]. However, the roles of ABA-responsive R2R3-MYB TFs involved in tanshinone biosynthesis remains largely unexplored.

In this study, we identified an ABA-responsive R2R3-MYB TF *SmMYB32*, as a positive regulator of tanshinone biosynthesis. Biochemical analysis demonstrated that SmMYB32 transcriptionally activated *SmCYP76AH3* and *SmCYP71D411* expression. In addition, SmPYL8 (an ABA receptor) was identified as an interaction partner of SmMYB32. SmPYL8 acted as a negative regulator in tanshinone biosynthesis. The interaction between SmPYL8 and SmMYB32 attenuates the transcriptional activity of *SmCYP76AH3* and *SmCYP71D411*. Taken together, this study uncovers new regulatory mechanism by which SmMYB32 integrates ABA signaling with tanshinone biosynthesis and provides new insights into metabolic engineering in *S. miltiorrhiza*.

## Results

### Isolation and characterization of SmMYB32

Previous studies have reported that tanshinone content could be increased by ABA treatment [16]. To further elucidate the mechanism by which ABA regulates the accumulation of bioactive ingredients in *S. miltiorrhiza*, transcriptome sequencing was performed [16]. In addition, tanshinones are primarily biosynthesized and accumulated in the roots. Based on the ABA-induced and tissue-specific transcriptomes data, we selected a candidate gene (TRINITY_DN352_c0_g1, which was designated as *SmMYB32* through blast) for further study (Fig. S1 and Table S1).

The full-length cDNA of *SmMYB32* (GenBank: KF059386.1) contains a 774-bp open reading frame (ORF), encoding a protein of 257 amino acids, with a calculated molecular weight of 28 kDa and a theoretical pI of 7.72. A phylogenetic tree constructed showed that SmMYB32 was most closely related to AtMYB44, AtMYB70, AtMYB73, and AtMYB77 and can be classified into the S22 subfamily (Fig. S2). Multiple-sequence alignment further revealed that SmMYB32 possesses a conserved R2R3 domain at its N-terminus (Fig. S3).

Members of the S22 MYB subfamily members have been reported to regulate the biosynthesis of bioactive compounds in various plant species, suggesting SmMYB32 may play a role in secondary metabolism in *S. miltiorrhiza*. Quantitative expression analysis revealed that *SmMYB32* was induced by ABA treatment and achieved the peak at 1 h with ~6-fold higher expression than the control (Fig. 1A). Additionally, tissue expression analysis indicated that *SmMYB32* exhibited higher expression in root, stem, and flower (Fig. 1B). Subcellular localization analysis using transient expression of SmMYB32-yellow fluorescent protein (YFP) in *Nicotiana benthamiana* epidermal cells indicated the nucleus localization pattern for SmMYB32 in contrast to the control (Fig. 1C).

**Figure 1:**
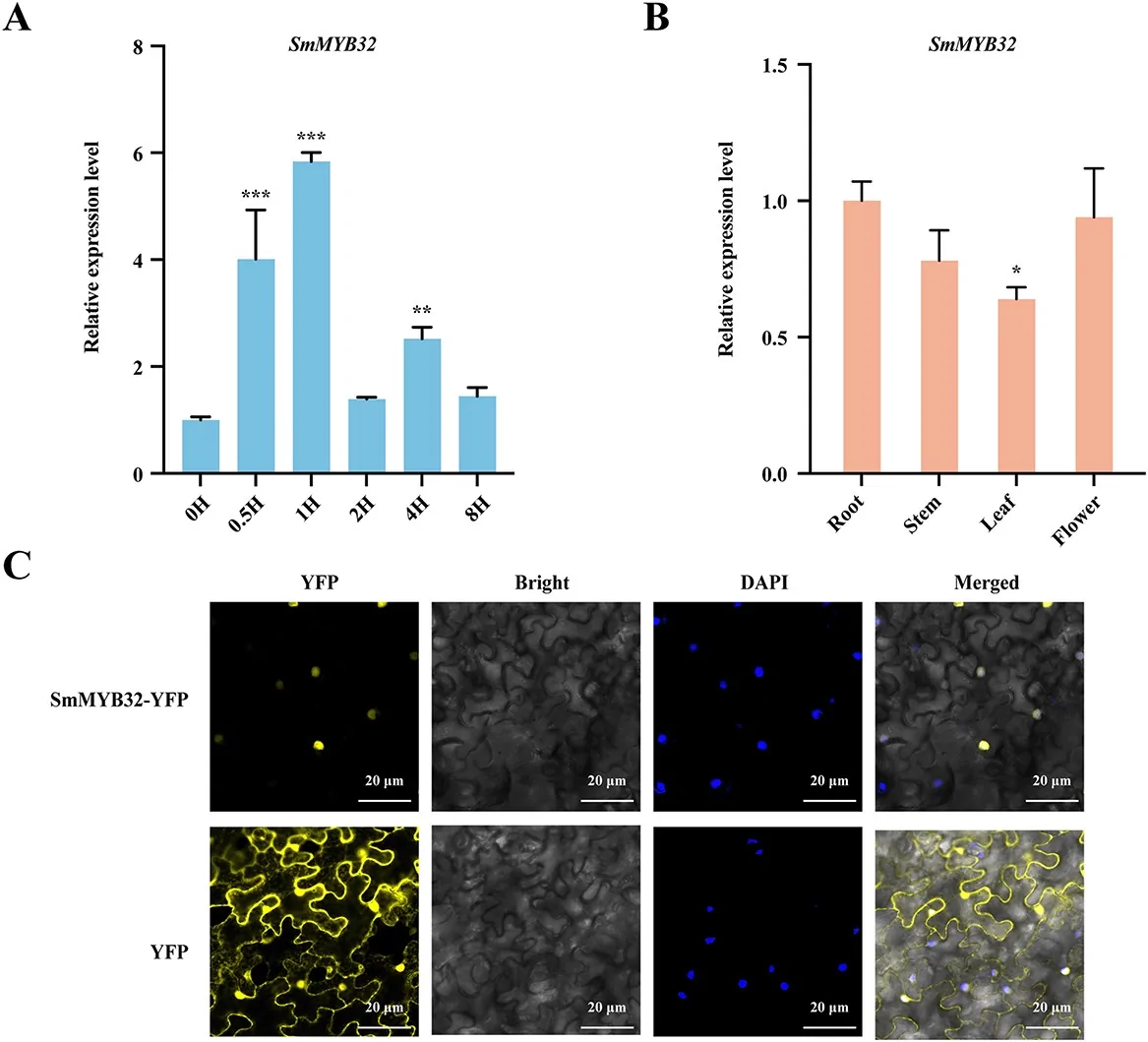
Expression patterns and subcellular localization of SmMYB32. (A) Expression level of *SmMYB32* after ABA induction. (B) Tissue expression profile of *SmMYB32*. *SmActin* served as the internal control. The values are means ± standard deviation (SD) of triplicate analyses. The significance levels were assessed using analysis of variance (ANOVA), ^*^P < 0.05, ^**^P < 0.01, ^***^P < 0.001. (C) Subcellular localization of SmMYB32. YFP was observed using the confocal microscope at 488 nm. YFP served as the control. Merged, overlay of the YFP, DAPI and Bright images. DAPI, 4′,6-diamidino-2-phenylindole. Bar, 20 μm.

### SmMYB32 positively regulated the biosynthesis of tanshinones

To investigate the role of SmMYB32 in *S. miltiorrhiza*, OE (Fig. S4A) and RNA interference (RNAi) (Fig. S4A) constructs were generated and transformed into *Agrobacterium* C58C1 for genetic transformation. A total of seven OE lines (39% positive rate) (Fig. S4B) and 19 RNAi lines (43% positive rate) were obtained (Fig. S4C). Based on the growth state and transcript levels of *SmMYB32*, three OE lines #4, #8, and #12 with higher expression and three RNAi lines #32, #37, and #38 with reduced expression compared with the control lines were selected for further analysis. High-performance liquid chromatography (HPLC) analysis was used to measure tanshinone content. The OE lines exhibited a darker red coloration than the controls, and total tanshinone content increased by ~3-, 1.5-, and 2.9-fold, respectively (Fig. 2A and C). Among these, OE#4 produced the highest yield of tanshinones, reaching 7.0 mg/g DW (dry weight). In contrast, *SmMYB32*-RNAi hairy roots displayed markedly lighter phenotype, and total tanshinone content was reduced by 70%, 50%, and 30%, respectively (Fig. 2B and D). To further investigate the role of SmMYB32 in ABA-induced tanshinone accumulation, the RNAi hairy roots were treated with 50 μM ABA. Total tanshinone content in the control lines increased by 1.76-fold after ABA treatment, whereas no obvious change was observed in RNAi lines. These results revealed that SmMYB32 mediated the ABA-induced accumulation of tanshinones (Fig. 2E and F; Fig. S5).

**Figure 2:**
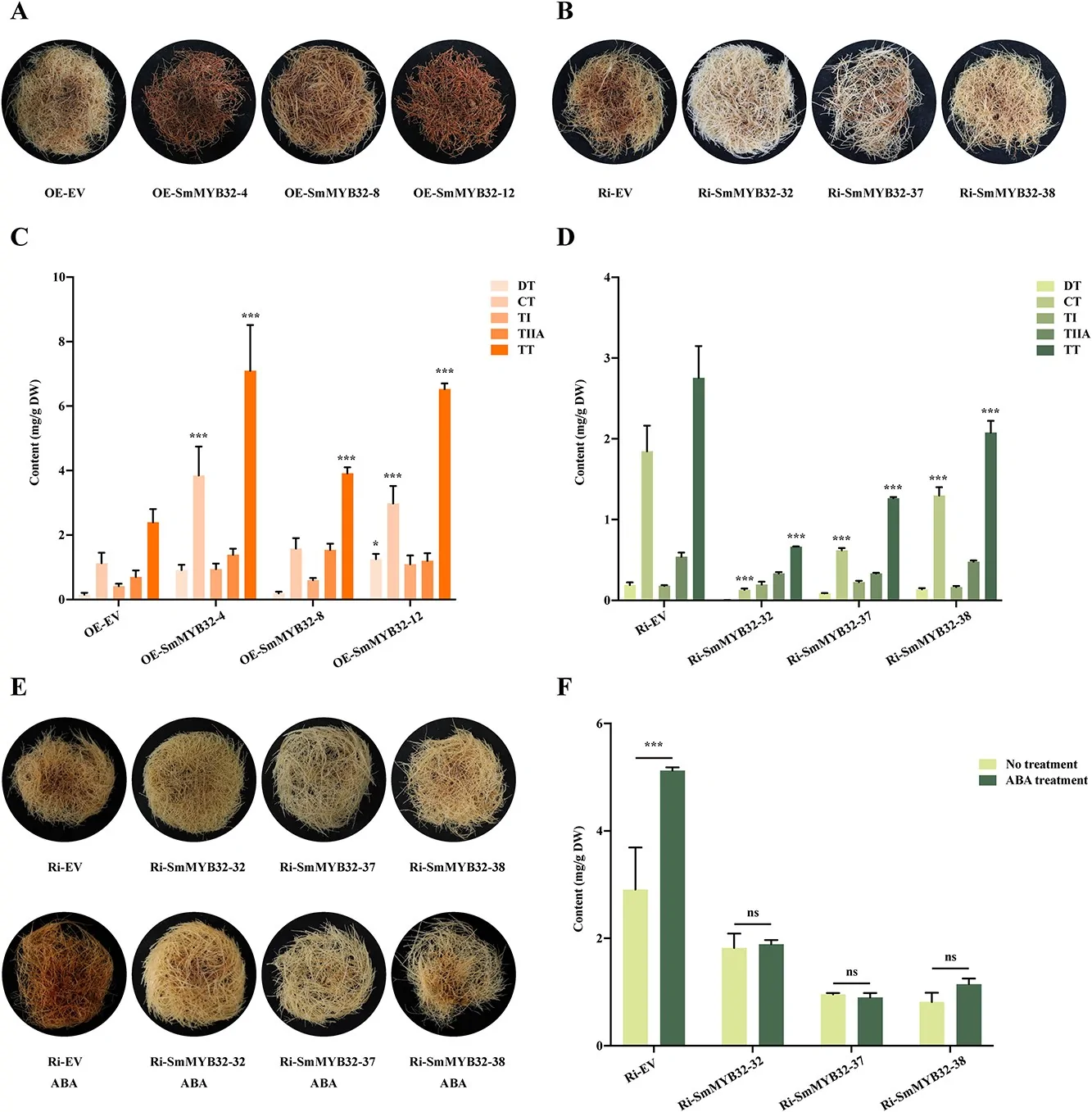
Tanshinone content in *SmMYB32* transgenic hairy roots. (A) Phenotype of *SmMYB32*-OE lines. (B) Phenotype of *SmMYB32*-RNAi lines. (C) Tanshinone content in *SmMYB32*-OE lines detected by HPLC. Asterisks denote significant difference by ANOVA (^*^P < 0.05, ^***^P < 0.001). DT, dihydrotanshinone I; CT, cryptotanshinone; TI, tanshinone I; TIIA, tanshinone IIA; TT, total tanshinones. (D) Analysis of tanshinone content in *SmMYB32*-RNAi lines (ANOVA: ^***^P < 0.001). (E) Phenotype of *SmMYB32*-RNAi lines under untreated and ABA-treated conditions. (F) Total tanshinone content in *SmMYB32*-RNAi lines following untreated and ABA-treated conditions. Asterisks denote significant difference by the Student’s *t*-test (ns, no significance; ^***^P < 0.001).

### SmMYB32 transcriptionally activates the expression of *SmCYP76AH3* and *SmCYP71D411*

The expression levels of genes involved in tanshinone biosynthesis were examined by quantitative real‑time PCR (qRT-PCR). In the tanshinone biosynthetic pathway, two CYP450s genes, including *SmCYP76AH3* and *SmCYP71D411*, were significantly upregulated in overexpression lines. In contrast, the expression of *SmDXR*, *SmGGPPS*, *SmCPS*, *SmKSL*, *SmCYP76AH3*, *SmCYP76AK1*, *SmCYP71D373*, *SmCYP71D375*, and *SmCYP71D411* was downregulated to varying degrees in RNAi lines (Fig. 3A and B). To further assess transcription activity, a dual luciferase (dual-LUC) assay was performed. Promoter sequences of tanshinone-biosynthetic genes were fused into the pGreen0800-LUC vector, with CaMV35S driving *REN* as an internal control to generate the reporter constructs, and SmMYB32 driven by the 35S promoter was used as the effector. The dual-LUC assay demonstrated that SmMYB32 transcriptionally activated the promoters of *SmCYP76AH3* and *SmCYP71D411* (Figs 3C and D and S6). Additionally, yeast one-hybrid (Y1H) assay showed that SmMYB32 binds to the MYB-binding site (MBS) located at –143 to –138 bp in the *SmCYP76AH3* promoter and –2345 to –2340 bp in the *SmCYP71D411* promoter (Fig. 3E and F). This interaction was further confirmed by electrophoretic mobility shift assay (EMSA) using purified GST-SmMYB32 recombinant protein and biotin-modified oligonucleotide probes containing the MBS sequences (Fig. 3G and H). Together, these results indicated that SmMYB32 directly binds to and transcriptionally activates *SmCYP76AH3* and *SmCYP71D411*.

**Figure 3:**
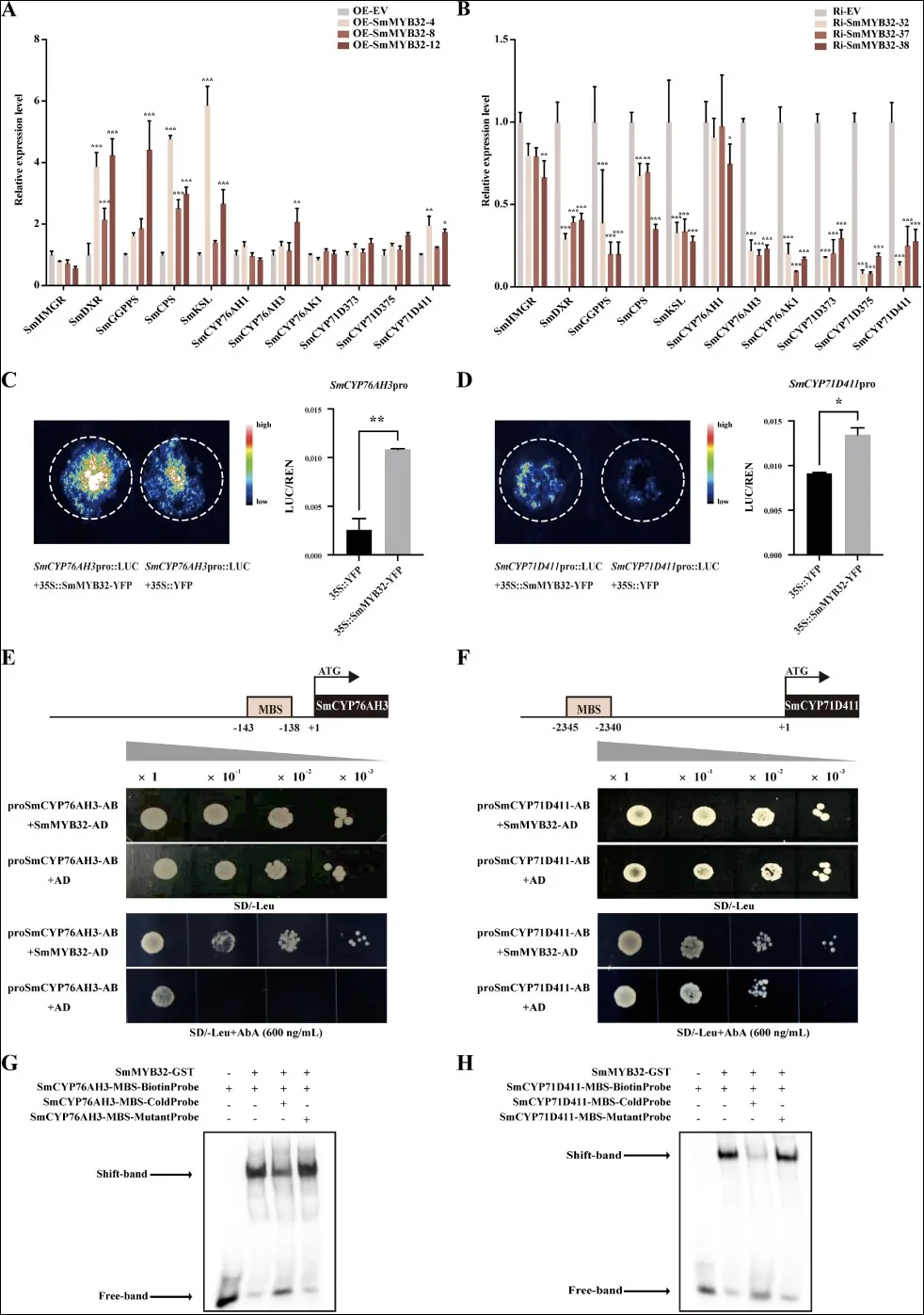
SmMYB32 transcriptionally activated the expression of *SmCYP76AH3* and *SmCYP71D411*. (A) Expression level of key enzyme genes in *SmMYB32*-OE hairy root lines. *SmActin* gene was used as the reference gene. The error bars represent ± SD of three independent biological replicates (ANOVA: ^*^P < 0.05, ^**^P < 0.01, ^***^P < 0.001). *SmHMGR*, 3-hydroxy-3-methylglutaryl-CoA reductase; *SmDXR*, 1-deoxy-D-xylulose-5-phosphate reductoisomerase; *SmGGPPS*, geranylgeranyl diphosphate synthase; *SmCPS*, copalyl diphosphate synthase; *SmKSL*, kaurene synthase like; Cytochrome P450 oxidase: *SmCYP76AH1*, *SmCYP76AH3*, *SmCYP76AK1*, *SmCYP71D373*, *SmCYP71D375*, *SmCYP71D411*. (B) Expression profiles of key biosynthesis genes in *SmMYB32*-RNAi lines. The detected genes are the same as those in (A), with the internal control and criteria for statistical significance consistent with those described in (A). (C) Dual-LUC assay showing that SmMYB32 activates the *SmCYP76AH3* promoter. Effector, 35S:YFP, 35S:SmMYB32-YFP; Reporter, pro*SmCYP76AH3*:LUC. The LUC:REN represented the relative luciferase activity. The REN served as internal control. All data presented here are means ±SD of three biological replicates. Statistical significance was determined by the Student’s *t*-test, ^**^P < 0.01. (D) Dual-LUC assay showing the activation of SmMYB32 on the *SmCYP71D411* promoter. The experimental design and criteria for statistical significance are as described in (C), ^*^P < 0.05. (E) Y1H assay detecting the binding of SmMYB32 to the MBS element in the *SmCYP76AH3* promoter. The plasmid pGADT7-SmMYB32 was cotransformed with pAbAi-*SmCYP76AH3*pro-MBS into Y1HGold yeast. Transformants were cultured on SD/-Leu (supplemented with AbA) for selection. (F) Y1H assay detecting the binding of SmMYB32 to the MBS element in the *SmCYP71D411* promoter. The experimental design and methods are the same as those described in (E). (G) EMSA results showed SmMYB32 bound to the labeled probes of *SmCYP76AH3*pro. Nonlabeled MBS element was used as the competitor. The mutant probes were without biotin labels. (H) EMSA assay validating the binding of SmMYB32 to the *SmCYP71D411* promoter. The experimental design and methods are as described in (G).

### SmMYB32 interacted with SmPYL8

To excavate potential interacting partners of SmMYB32 and elucidate its regulatory mechanism, a yeast two-hybrid (Y2H) screen was conducted using pGBKT7-SmMYB32 as the bait against a whole-tissue cDNA library of *S. miltiorrhiza*. A PYL member designated as SmPYL8 (GenBank: XM_057942659.1) was identified. PYR/PYL/RCAR proteins function as intracellular ABA receptors and play critical roles in ABA signal perception and transduction via the PP2C-SnRK2 pathway. Using Arabidopsis PYL proteins as queries, we identified 11 PYL homologs in the *S. miltiorrhiza* genome. Phylogenetic analysis of the PYLs from *Arabidopsis thaliana* and *S. miltiorrhiza* revealed that SmPYL8 is closely related to AtPYL7, AtPYL8, AtPYL9, and AtPYL10 (Fig. S7A). Heatmap analysis indicated that *SmPYL8* expression decreased following ABA treatment (Fig. S7B and Table S2), which was further confirmed by qRT-PCR (Fig. S8).

Y2H assay showed that yeast cells harboring pGBKT7-SmMYB32 and pGADT7-SmPYL8 grew normally on selective SD/-Leu/-Trp/-His/-Ade medium, whereas those coexpressing pGBKT7-SmMYB32 and pGADT7-EV did not, indicating the interaction between SmMYB32 and SmPYL8. The interaction strength was enhanced upon ABA treatment (Fig. 4A). *In vitro* pull-down assay further confirmed a direct interaction between SmMYB32 and SmPYL8, as evidenced by the pull-down of SmPYL8-His by MYB32-GST but not by GST protein (Fig. 4B). In addition, bimolecular fluorescence complementation (BiFC) assays showed strong fluorescence signal in *N. benthamiana* leaves coexpressing SmMYB32-nYFP and SmPYL8-cYFP, whereas no signal was detected in the negative controls (Fig. 4C). Together, these results demonstrate SmMYB32 physically interact with SmPYL8 both *in vitro* and *in vivo*.

**Figure 4:**
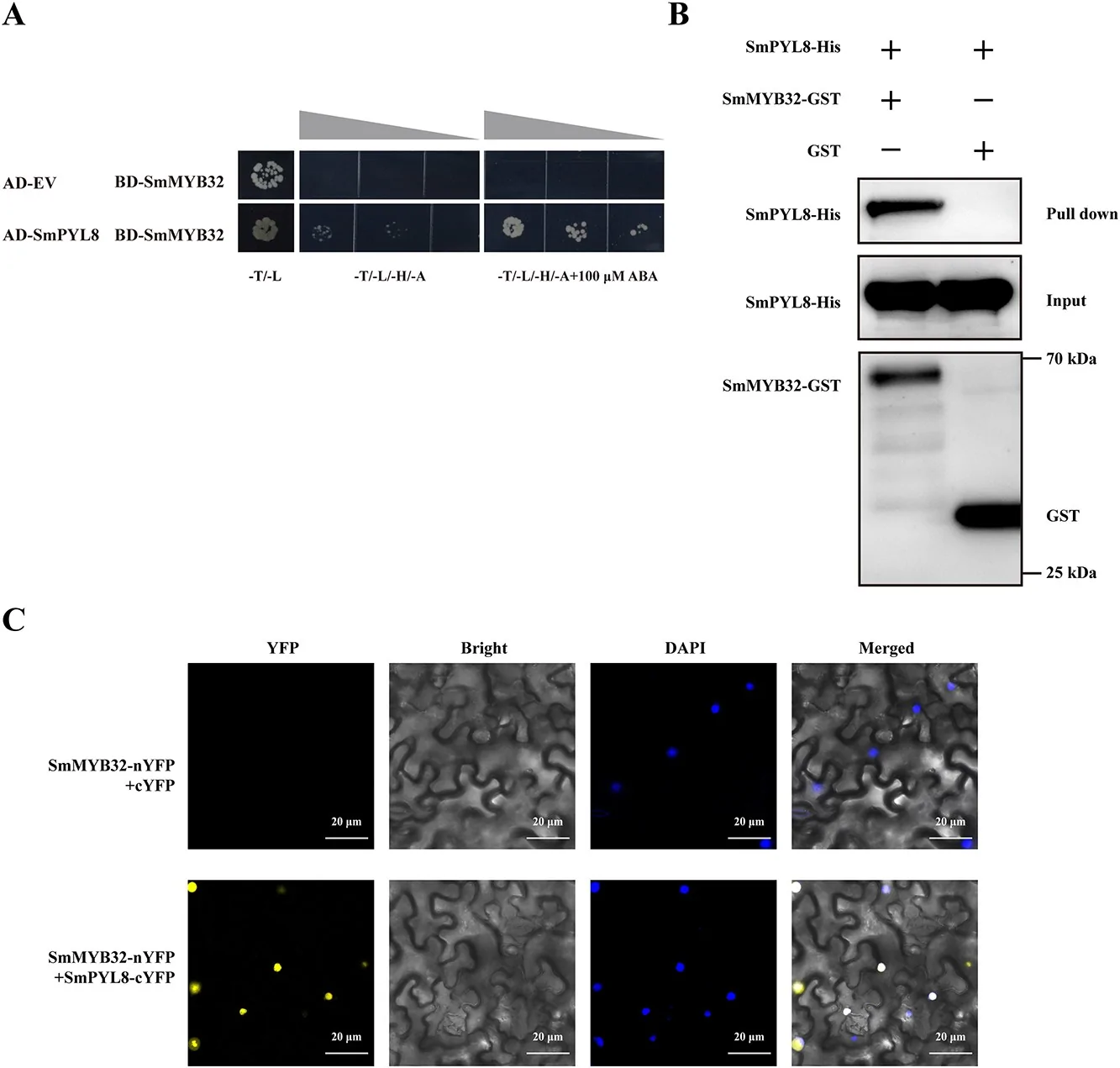
SmMYB32 interacted with SmPYL8 *in vitro* and *in vivo*. (A) Y2H assay showing that SmMYB32 interacted with SmPYL8. AH109 yeast cells cotransformed with pGBKT7-SmMYB32 and pGADT7-SmPYL8 grew normally in SD/-Trp/-Leu/-His/-Ade medium, and the addition of 100 μM ABA promoted the growth of the cotransformed strain. (B) *In vitro* pull-down assay showed that SmMYB32 interacted with SmPYL8. Recombinant SmPYL8-His was incubated with SmMYB32-GST and analyzed by immunoblot using an anti-His antibody or anti-GST antibody. (C) BiFC analysis of the interaction between SmPYL8 and SmMYB32. The YFP was only observed when SmMYB32-nYFP was coexpressed with SmPYL8-cYFP. Bar, 20 μm.

### SmPYL8 negatively affected tanshinone biosynthesis and attenuated the transcriptional activity of SmMYB32

To explore the role of SmPYL8 on tanshinone biosynthesis, OE hairy root lines (OE-*SmPYL8*) were generated (Fig. S9A). Hairy roots of *SmPYL8*-OE lines #3, #7, and #10, exhibiting robust growth and high *SmPYL8* expression levels (Fig. S9B and D), were selected for further analysis. HPLC analysis revealed that total tanshinone content decreased from 3.07 mg/g DW to 1.24 mg/g DW, with line #3 exhibiting the lowest content of tanshinones (Fig. 5A and C). qRT-PCR analysis demonstrated that expression level of *SmGGPPS*, *SmCYP76AH3*, *SmCYP76AK1*, and *SmCYP71D411* were significantly decreased compared with control (Fig. 5E). Subsequently, *SmPYL8*-RNAi (Ri-*SmPYL8*) hairy root lines were successfully generated. Lines #7 and #12, which showed vigorous growth and markedly reduced *SmPYL8* transcript levels, were selected for further characterization (Fig. S9A, C, and  E). HPLC analysis revealed that total tanshinone content increased from 0.21 mg/g DW in the control to 1.23 mg/g DW, with line #12 showing the highest accumulation (Fig. 5B and D). Furthermore, qRT-PCR analysis demonstrated that the transcript levels of *SmHMGR*, *SmCYP76AH3*, *SmCYP76AK1*, and *SmCYP71D411* were significantly upregulated relative to the control (Fig. 5F).

**Figure 5:**
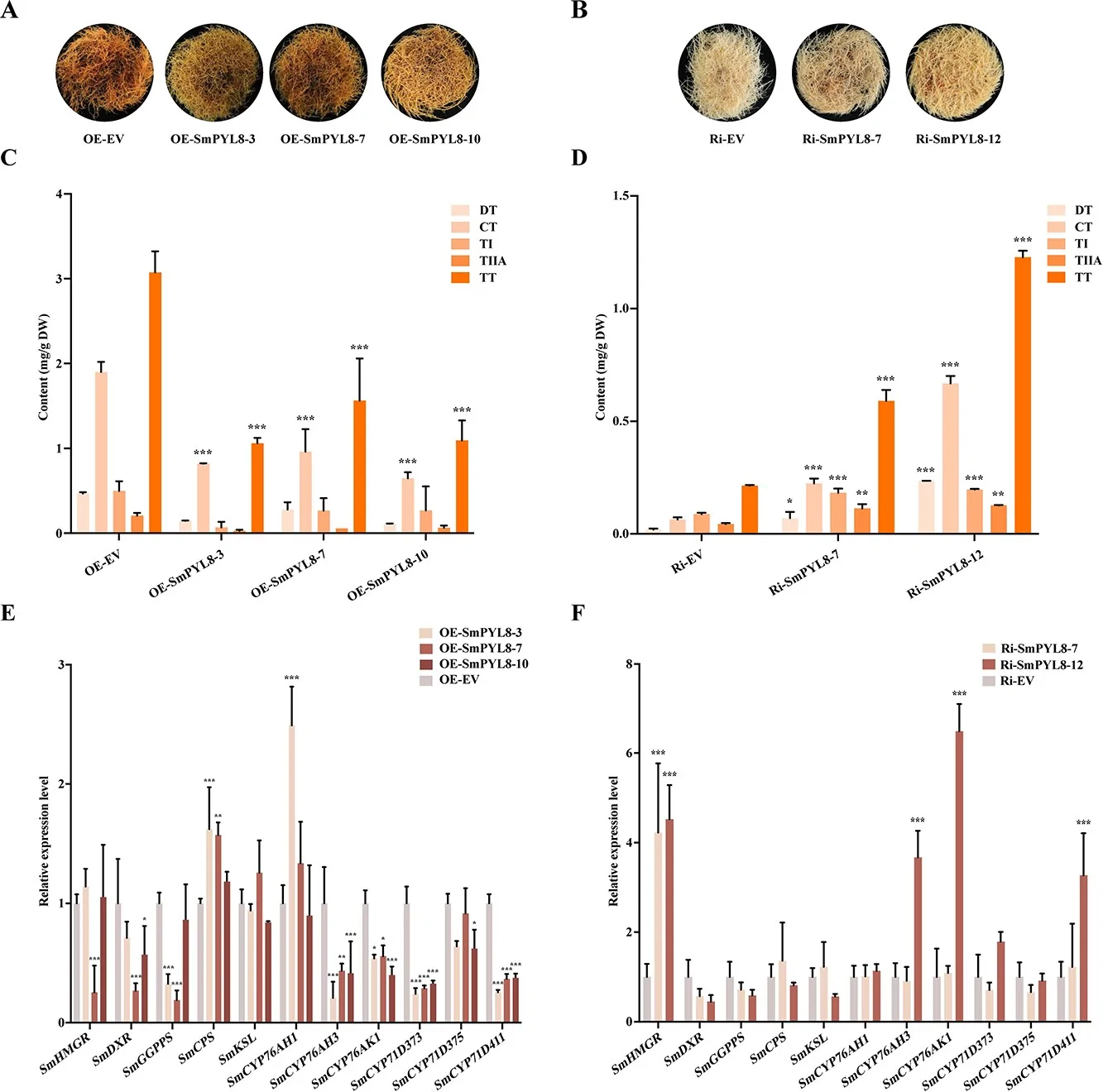
SmPYL8 negatively regulated the biosynthesis of tanshinones. (A) Phenotype of *SmPYL8*-OE hairy roots. (B) Phenotype of Ri*-SmPYL8* hairy roots. (C) Tanshinone content in *SmPYL8*-OE hairy roots determined by HPLC. Asterisks denote significant difference by ANOVA (^***^P < 0.001). (D) HPLC analysis of tanshinones in Ri*-SmPYL8* lines. (ANOVA: ^*^P < 0.05, ^**^P < 0.01, ^***^P < 0.001). (E) Expression levels of key biosynthetic genes in *SmPYL8*-OE hairy roots, normalized to *SmActin*. The error bars represent ±SD of the three independent replicates (ANOVA: ^*^P < 0.05, ^**^P < 0.01, ^***^P < 0.001). (F) Expression levels of key biosynthetic genes in Ri*-SmPYL8* hairy roots, normalized to *SmActin*. The error bars represent ±SD of the three independent replicates (ANOVA: ^*^P < 0.05, ^**^P < 0.01, ^***^P < 0.001).

Given that SmPYL8 interacted with SmMYB32, and that both influence tanshinone biosynthesis, we conducted a dual-LUC assay to determine whether SmPYL8 antagonizes the transcriptional activity of SmMYB32 on its target promoters. Three effector constructs (35S:YFP, 35S:SmMYB32-YFP, and 35S:SmPYL8-YFP) and the reporter constructs containing the promoters of *SmCYP76AH3* and *SmCYP71D411* fused to LUC were employed in the assay. The results suggested that coexpression of SmMYB32 with SmPYL8 significantly reduced the relative LUC activity of *SmCYP76AH3pro* and *SmCYP71D411pro* compared with SmMYB32 alone (Fig. 6). These findings indicated that SmPYL8 attenuates the transcriptional activation activity of SmMYB32 on target genes.

**Figure 6:**
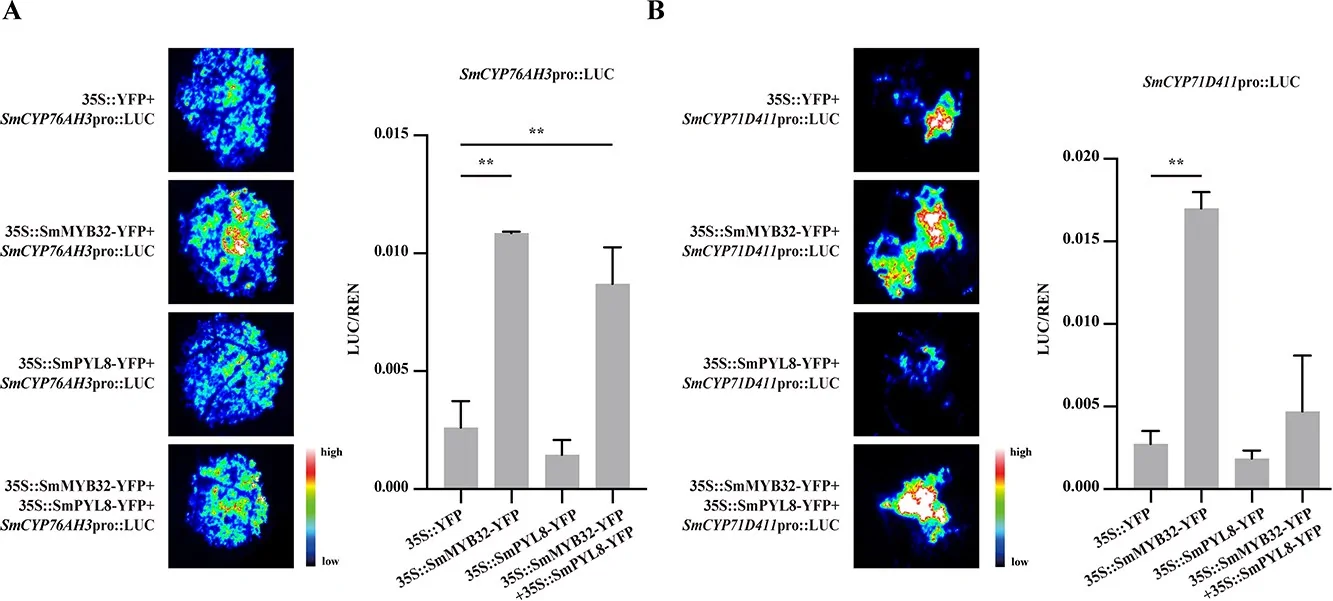
SmPYL8 repressed the transcriptional activity of *SmMYB32*. (A) Dual-LUC assay showing that SmPYL8 inhibits the transcriptional activity of SmMYB32. Effector, 35S:YFP, 35S:SmMYB32-YFP, 35S:SmPYL8-YFP; Reporter, pro*SmCYP76AH3*:LUC. The LUC:REN represented the relative luciferase activity. The REN served as internal control. Error bars indicating ±SD. Statistical significance was determined by the ANOVA, ^**^P < 0.01. (B) Using pro*SmCYP71D411*:LUC as the reporter gene and REN as the internal control revealed that the effector SmPYL8-YFP significantly inhibited the transcriptional activity of SmMYB32-YFP (ANOVA: ^**^P < 0.01).

## Discussion

### SmMYB32 is a positive regulator-mediated ABA promoting tanshinone biosynthesis

The phytohormone ABA plays essential role in various physiological processes in plants such as growth and development [29–31]. Besides, ABA can act as exogenous elicitor to enhance the production of secondary metabolites in traditional herbs. For instance, ABA treatment improved the artemisinin content in *Artemisia annua* [32]*.* Similarly, ABA application significantly elevated the accumulation of tanshinones in *S. miltiorrhiza* hairy roots or original plants [13, 16]. To elucidate the underlying regulatory mechanism, deep transcriptome sequencing of 50 μM ABA-induced hairy roots of *S. miltiorrhiza* were conducted previously and multiple TFs were identified [16]. Candidate TFs were screened based on their significantly elevated expression (FPKM >200 and a >5-fold induction) as well as the tissue-specific accumulation pattern of tanshinone biosynthesis. Our previous studies characterized ABA-responsive bZIP TFs for functional characterization. For example, SmbZIP1 played a negative role in tanshinone accumulation [33]. SmbZIP4 was established as a repressor of tanshinone biosynthesis by directly targeting *SmGGPPS* and *SmCYP76AK1* in both hairy roots and transgenic plants [13], whereas SmbZIP5 functioned as a positive regulator [11]. At present, MYB TFs that respond to ABA induction remain largely unexplored. In this study, *SmMYB32*, an ABA-responsive R2R3-MYB TF, was identified and functionally characterized. Phylogenetic tree analysis classified SmMYB32 into S22 subfamily. Overexpression of *SmMYB32* in *S. miltiorrhiza* hairy roots produced higher content of tanshinones, with the highest content reaching 3-fold that of control. qRT-PCR analysis revealed that expression level of key genes in MEP pathway and downstream pathway such as *SmDXR*, *SmDXS2*, *SmGGPPS*, *SmCPS*, *SmKSL*, *SmCYP76AH3*, *SmCYP71D373*, *SmCYP71D375*, and *SmCYP71D411* were obviously upregulated in OE lines, and downregulated in RNAi lines. In combination with dual-LUC, Y1H and EMSA assays further revealed that SmMYB32 transcriptionally activated *SmCYP76AH3* and *SmCYP71D411* by binding to MBS element in their promoters. Moreover, ABA treatment failed to induce tanshinone accumulation in RNAi lines, while the control lines yielded a significant increase. These findings demonstrated that SmMYB32 functions as a positive regulator in tanshinone biosynthesis via the ABA signaling pathway.

### *SmMYB32* indirectly affected phenolic acid biosynthesis

Phenolic acids are another major class of bioactive ingredients in *S. miltiorrhiza*, which are produced via two distinct biosynthetic routes. Key enzymes include phenylalanine ammonia-lyase (PAL), cinnamic acid 4-hydroxylase (C4H), and 4-coumarate:CoA ligase (4CL) in the phenylpropane pathway, as well as tyrosine aminotransferase (TAT) and 4-hydroxyphenylpyruvate reductase (HPPR) in the tyrosine-derived pathway. Intermediates from these two pathways are utilized by the key enzyme CYP98A14 to form rosmarinic acid (RA). Specific reaction from RA to SalB has not been fully elucidated [33, 34]. In this study, phenolic acid content decreased in OE lines (Fig. S10A), and increased in RNAi lines (Fig. S10B), suggesting an inverse relationship with tanshionone accumulation. qRT-PCR result showed that expression level of key biosynthetic genes such as *SmC4H*, *SmHPPR*, *Sm4CL*, *SmRAS*, and *SmCYP98A14* were downregulated in OE lines (Fig. S10C), and RNAi lines showed no obvious transcriptional change except for *SmTAT* (Fig. S10D). We speculate that this effect is mainly attributed to end-product feedback repression. Suppression of *SmMYB32* led to flux redistribution, resulting in increased accumulation of phenolic acids, whereas elevated level of end-products represses the transcription of biosynthetic pathway genes such as *SmHPPR*, *SmRAS*, and *SmCYP98A14* to maintain metabolic homeostasis. In addition, dual-LUC assay showed that SmMYB32 has no significant regulatory effect on these biosynthetic genes. These results suggested that SmMYB32 is not directly involved in phenolic acid biosynthesis.

### SmPYL8 physically interacted with SmMYB32 to repress its transcriptional activity

To further investigate the regulatory mechanism of SmMYB32 in tanshinone and phenolic acid biosynthesis, a Y2H screen using pGBKT7-SmMYB32 as the bait identified several interaction partners including ABA receptor SmPYL8. Transgenic analysis showed that overexpression of *SmPYL8* resulted in decreased tanshinone content, accompanied by reduced expression level of *SmGGPPS*, *SmCYP76AH3*, *SmCYP76AK1*, and *SmCYP71D411*. To assess the impact of SmMYB32-SmPYL8 interaction on target gene expression, effector combined with pHB-SmMYB32-YFP and pHB-SmPYL8-YFP were used to conduct dual-LUC assay. The result showed that the activities of the *SmCYP76AH3* and *SmCYP71D411* were repressed compared with using pHB-SmMYB32-YFP alone, suggesting that SmPYL8 weakened the transcriptional activity of SmMYB32 on its target genes. Interestingly, the phenolic acid content increased to an average of 17.7 mg/g DW in OE-*SmPYL8* hairy root lines, which was 1.57 times as that in control (Fig. S11A), while expression of biosynthetic pathway genes did not change significantly (Fig. S11B), further supporting indirect regulation.

As ABA receptors, RCARs/PYR1/PYLs acted as core components in ABA signaling. A systematic analysis of ABA receptors identified 11 PYL members in *S. miltiorrhiza*. Here, the role of SmPYL8 in tanshinone and phenolic acid biosynthesis has been investigated. Interestingly, S22 MYB members in *Arabidopsis* (AtMYB44, AtMYB70, AtMYB73, and AtMYB77) interact with AtPYL9, suggesting that S22 subfamily members may have overlapping functions and potential genetic redundancy [35]. S22 MYBs in *Arabidopsis* are also known to primarily participate in response to biotic and abiotic stress. For example, AtMYB44 enhanced resistance to pathogens by interacting with AtWRKY70 to suppress jasmonic acid-mediated defense and activate salicylic acid-mediated defense. It also participates in ABA-mediated stomatal closure and improves salt and drought tolerance [36–38]. AtMYB77 interacts with AtUVR8 to regulate lateral root growth, indirectly influencing root responses to biotic stresses like root rot [39]. These findings suggest SmMYB32 may integrate stress signaling with secondary metabolism in *S. miltiorrhiza*. Therefore, further investigation of function of S22 subfamily in *S. miltiorrhiza* is warranted.

### A working model for SmMYB32 function in tanshinone biosynthesis

Multiple MYBs have been reported to participate in tanshinone biosynthesis in *S. miltiorrhiza*; however, ABA-responsive R2R3-MYB members remain poorly characterized. Based on our findings, we proposed a model in which SmMYB32 functions as a key regulating regulator linking ABA signaling to tanshinone biosynthesis. ABA-responsive *SmMYB32* promoted tanshinone biosynthesis by activating the downstream pathway genes *SmCYP76AH3* and *SmCYP71D411* through binding to MBS *cis*-elements in their promoter. As an ABA receptor, SmPYL8 physically interacts with SmMYB32 and represses its transcriptional activity on targets, leading to decreased tanshinone content in *S. miltiorrhiza*. Under stress conditions, ABA levels rapidly increase, leading to suppression of *SmPYL8* expression and concurrent upregulation of *SmMYB32*. Subsequently, the inhibitory effect of SmPYL8 on SmMYB32 is alleviated, enabling SmMYB32 to activate the transcription of *SmCYP76AH3* and *SmCYP71D411* and thereby enhance tanshinone production (Fig. 7).

**Figure 7:**
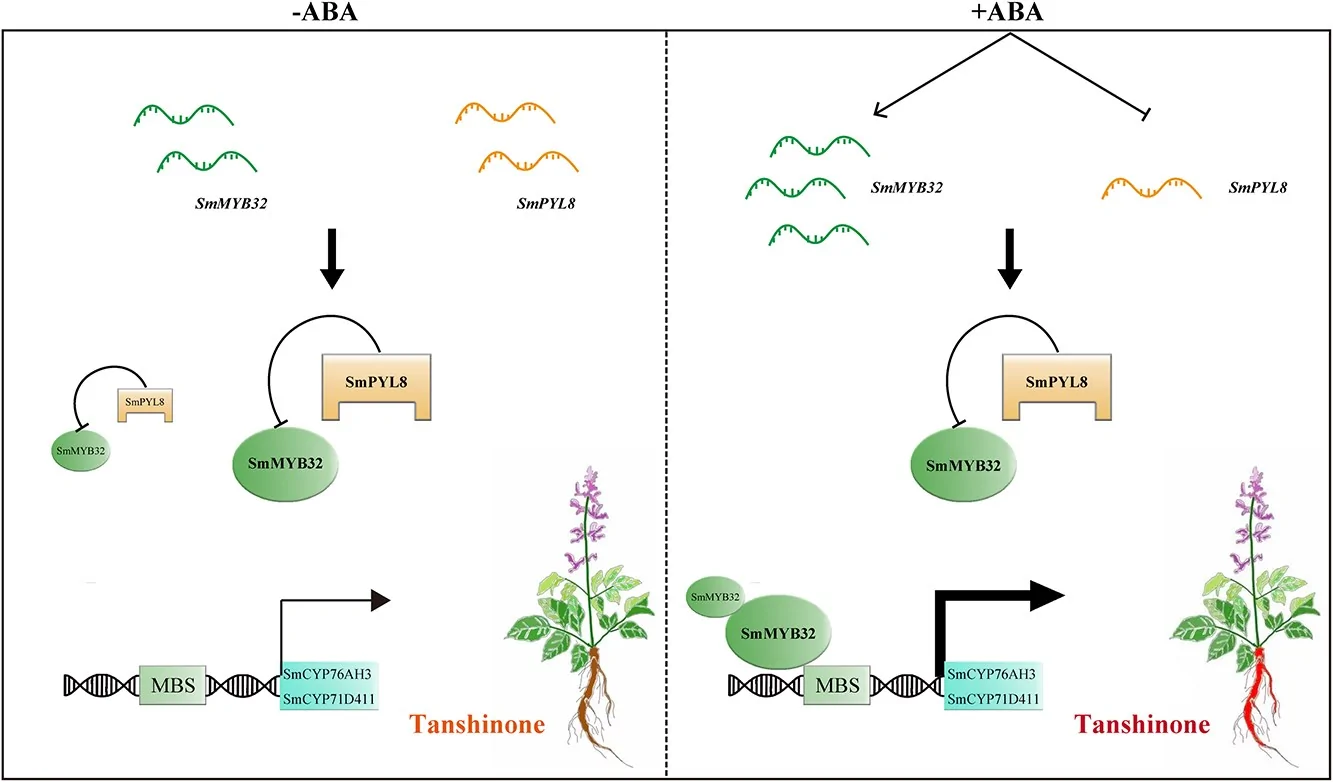
SmMYB32 interacts with SmPYL8 to regulate the biosynthesis of tanshinones. In the absence of ABA, SmPYL8 inhibits the transcriptional activity of SmMYB32, resulting in reduced expression of *SmCYP76AH3* and *SmCYP71D411* and decreased tanshinone content. In the presence of ABA, *SmMYB32* expression is induced while *SmPYL8* expression is suppressed. Under these conditions, SmPYL8’s inhibitory effect on SmMYB32 is alleviated. SmMYB32 then binds to the MBS element in the promoter and transcriptionally activates the expression of *SmCYP76AH3* and *SmCYP71D411*, thereby promoting the accumulation of tanshinones. Arrows indicate promotion; ‘┴’ denotes repression; double helices represent DNA; single helices represent RNA; circles or polygons represent proteins; MBS refers to the MYB TF-binding site.

## Materials and methods

### Plant materials and ABA treatment

*S. miltiorrhiza* seedlings used for tissue expression analysis were grown in the greenhouse at Zhejiang Chinese Medical University under the culture condition of 25°C, 16 h light/8 h dark. Sterile seedlings of *S. miltiorrhiza* were cultured on Murashige and Skoog (MS) medium under the same condition. The acquisition process of C58C1 hairy roots is in accordance with our previous study [40]. Well-developed C58C1 hairy roots were inoculated into 100 mL of 1/2 MS medium and cultured at 25°C with shaking at 120 rpm in darkness. After 45 days, the hairy roots were treated with ABA at a final concentration of 50 μM, with sampling at 0, 0.5, 1, 2, 4, and 8 H [16]. *N. benthamiana* were grown under conditions described previously [19].

### RNA extraction and qRT-PCR analysis

Total RNA was extracted from 2-year-old *S. miltiorrhiza* plants grown in a greenhouse. RNA was isolated from the root, stem, leaf, and flower of *S. miltiorrhiza* plants using the plant RNA extraction kit (Vazyme) according to the manufacturer’s instructions. First-strand cDNA was synthesized following the reverse transcription protocol (Vazyme). RNA extraction and cDNA synthesis for hairy roots were performed using the same procedure. qRT-PCR analysis was performed as described previously [41, 42]. *SmActin* was used as the internal reference gene. The relative gene expression levels were calculated using the 2^-ΔΔCT^ method. The primer sequences are listed in Table S3.

### Bioinformatics analysis of SmMYB32

The sequences of 125 MYB TFs from *A. thaliana* (AtMYB1-AtMYB125) were retrieved from the TAIR database (https://www.arabidopsis.org). A phylogenetic tree of SmMYB32 and AtMYBs was constructed using MEGA software with Neighbor-Joining method [43]. The online tool iTOL (https://itol.embl.de) was used to embellish the phylogenetic tree. Multiple-sequence alignment between SmMYB32 and AtMYBs was performed using DNAMAN.

### Subcellular localization

The ORF of *SmMYB32* was cloned into the vector pHB-YFP between *Bam*HI and *Spe*I to obtain the SmMYB32-YFP fusion protein driven by the CaMV35S. Recombinant vector pHB-*SmMYB32*-YFP and the control vector pHB-YFP were separately transformed into *Agrobacterium* GV3101, and then infiltrated into 1-month-old *N. benthamiana* leaves. The infiltrated plants were cultured in darkness for 24 h and then transferred in light condition for another 24 h. Leaves were stained with 10 mg/mL 4′,6-diamidino-2-phenylindole (DAPI) for 2–3 h before observation. YFP fluorescence was detected using laser confocal microscope (Zeiss) [13, 44]. YFP fluorescence was excited at 488 nm and DAPI fluorescence at 405 nm.

### Plasmid construction and genetic transformation

The coding region of *SmPYL8* was cloned into the pCAMBIA2306-Flag to yield the recombinant vector pCAMBIA2306-*SmPYL8*-Flag [11]. pHB-*SmMYB32*-YFP and pCAMBIA2306-*SmPYL8*-Flag were respectively transformed into *Agrobacterium* C58C1 and then was used to obtain the overexpression lines. For gene silencing lines, 150 to 250bp gene-specific fragments from *SmMYB32* and *SmPYL8* were inserted into the pFGC5941 vector and transformed into *Agrobacterium* C58C1 [11]. Hairy root transformation was performed as described previously [42]. Hairy root DNA was extracted using the CTAB method and was verified by PCR using gene-specific primers (Table S3).

### High-performance liquid chromatography analysis of tanshinones

Equal amounts of transgenic and control hairy roots were cultured in 100 mL of 1/2 MS medium for 45 days, then collected and dried in a 55°C oven [21]. Samples were ground into powder. For tanshinone extraction, 50 mg of sample powder was dissolved in 16 mL of methanol: dichloromethane (3:1) and sonicated for 60 min. The supernatant was rotary evaporated at 55°C and redissolved in 2 mL methanol. After filtration through a 0.22μm filter, samples were analyzed using Agilent 1260 Infinity II HPLC system. Tanshinone content was detected using a C-18 reversed-phase silica column, following the method previously described [19, 45].

### Dual-luciferase assay

The promoter sequences of key enzymes in tanshinone biosynthesis pathway were cloned into the pGreen0800 vector and transformed into the GV3101 (pSoup). Effector constructs (pHB-SmMYB32-YFP and pHB-SmPYL8-YFP) were also transformed into the *Agrobacterium* GV3101. pHB-YFP was designated as the negative control. Reporter (pGreen0800-pro) and Effector (pHB) were mixed in a 1:1 ratio and infiltrated into *N. benthamiana* leaves. After 48 h of incubation, fluorescent expression was measured using a chemiluminescence instrument. The final concentration of potassium fluorescein is 1 mM. Luciferase activity was measured as described previously [27, 44].

### Y1H assay

The promoters of *SmCYP76AH3* and *SmCYP71D411* were cloned into pAbAi vector to obtain and linearized by *Bst*BI restriction enzyme, then transformed into Y1H Gold yeast competent cells and painted onto an SD/-Ura plate. The coding sequence (CDS) of *SmMYB32* was constructed into pGADT7 vector and transformed into yeast competent cells containing linearized plasmids of pAbAi-*proSmCYP71D411* and *proSmCYP76AH3*, respectively. The positive monoclonal clone was selected on SD/−Leu plates with Aureobasidin A and cultured at 30°C for 3–5 days [46].

### Electrophoretic mobility shift assay

The CDS of *SmMYB32* was constructed into pGEX4T-1 vector to generate SmMYB32-GST vector, and transferred into the Rosetta (DE3) strain. Recombinant protein was expressed and induced with 0.5 mM isopropyl β-D-1-thiogalactopyranoside (IPTG) and purified according to the instructions [11]. The probes were synthesized with 3′-end biotin labeling. Unlabeled cold probes and mutant probes were used as competitors. The purified protein was first incubated with binding buffer for 10 min to allow for nonspecific binding, followed by addition of probes for 15 min to enable specific binding. Samples were separated by 10% nondenaturing gel electrophoresis, transferred onto nylon membranes, and the migration bands were visualized using a chemiluminescence instrument [42].

### Y2H verification

The ORFs of *SmPYL8* and *SmMYB32* were cloned into vectors pGADT7 and pGBKT7, respectively, and cotransformed into AH109 yeast cells. Transformants were plated on SD/-Leu/-Trp plates and incubated at 30°C for 2–4 days. Cotransformants containing pGADT7 and pGBKT7-SmMYB32 were used as the negative control. The monoclonal clone was diluted in sterile water and cultured on SD/-Leu/-Trp/-His/-Ade plates, as well as the same medium supplemented with 100 μM ABA, followed by incubation in a 30°C incubator for 3–5 days [13].

### Bimolecular fluorescence complementation assay

*SmPYL8* and *SmMYB32* were cloned into pXY104-cYFP and pXY106-nYFP vectors, respectively, and were individually transformed into *Agrobacterium* GV3101. *Agrobacterium* carrying pXY104-*SmPYL8*-cYFP or pXY104-cYFP were mixed with *Agrobacterium* carrying pXY106-*SmMYB32*-nYFP at a 1:1 ratio and incubated at 28°C for 2–4 h. After infiltration into *N. benthamiana* and incubation for 48 h, YFP fluorescence and DAPI staining were observed under laser confocal microscopy [19].

### Pull-down assay

The pCold-SmPYL8*-*His construct was generated and transformed into Rosetta (DE3) strain. Expression of SmPYL8-His was induced using 0.5 mM IPTG and the protein was purified according to [27]. For the pull-down assay, purified SmPYL8-His and SmMYB32-GST proteins were sequentially incubated with 100 μL of GST magnetic beads (Sangon Biotech) as the experimental group, beads incubated with GST protein alone served as the negative control. Both groups were incubated overnight at 4°C. The supernatant was collected as the input sample, and the beads were washed five times with washing buffer. Protein loading buffer was added to both the input and bead samples, followed by denaturation at 99°C for 10 min. Western blot was performed as previously described [42]. using anti-GST and anti-His (TransGen Biotech) antibodies to detect target proteins.

## Supplementary Material

Web_Material_uhag186

## Data Availability

All data generated in the present study are included in this published article and its supplementary data files.
