# Supplementary material for: The ABA-responsive transcription factor SmMYB32 positively modulates tanshinone biosynthesis in Salvia miltiorrhiza
Source: Hortic Res. 2026 Nov 12;13(10):uhag186. doi: 10.1093/hr/uhag186 (PMC13724252; doi:10.1093/hr/uhag186)
Supplement: Supplementary file 1 [file web_material_uhag186.docx]

**Supplementary Information**

**Fig. S1 Expression of SmMYB32 in ABA-induced and tissue transcriptome.** 0H, 0.5H, 1H, 2H, 4H, 8H represent different time points following ABA treatment. F, flower; S, stem; L, leaf; R, root. Bluer colors indicate lower relative values; redder colors indicate higher relative values.

**Fig. S2 Phylogenetic tree construction of SmMYB32.** Phylogenetic analysis of SmMYB32 and AtMYBs. Amino acid sequences of AtMYBs and SmMYB32 were aligned and a phylogenetic tree was constructed and visualized using the Neighbor-Joining method through MEGA11 software. The statistical reliability of individual nodes was assessed by bootstrap analyses with 1000 replications.

**Fig. S3 Conserved domain analysis of SmMYB32.** Multiple sequence alignment of the deduced SmMYB32 protein with several AtMYBs. Represented R2R3 domain was highlighted.

**Fig. S4 Vector construction and PCR detection for SmMYB32.** (A) Overexpression (pHB) and RNAi (pFGC5941) vector construction of SmMYB32. (B) PCR detection of *SmMYB32* overexpression lines. (C) PCR detection of *SmMYB32* silencing lines. “M” represents DNA marker; “-” represents the negative control without DNA; “+” represents the positive control with added plasmid DNA.

**Fig. S5 Detection of DT, CT, TI, and TIIA content in *SmMYB32*-RNAi lines under untreated and ABA-treated conditions.** DT, dihydrotanshinone I; CT, cryptotanshinone; TI, tanshinone I; TIIA, tanshinone IIA; TT, total tanshinones. Asterisks denote significant differences determined by Student's *t*-test, ****P <* 0.001.

**Fig S6 Dual-luciferase reporter assay detecting the effect of SmMYB32 on tanshinone-biosynthesis genes.** Effector, 35S:YFP, 35S:SmMYB32-YFP; Reporter, pro*SmHMGR*:LUC, pro*SmDXR*:LUC, pro*SmGGPPS*:LUC, pro*SmCPS*:LUC, pro*SmKSL*:LUC, pro*SmCYP76AH1*:LUC, pro*SmCYP76AK1*:LUC, pro*SmCYP1D373*:LUC, pro*SmCYP1D375*:LUC. The LUC: REN represented the relative luciferase activity. The REN served as internal control. All data presented here are means ±SD of three biological replicates, with statistical significance was determined by the Student’s *t*-test, **P* < 0.01, ***P* < 0.01.

**Fig. S7 Phylogenetic tree and expression pattern analysis of PYL members in *S. miltiorrhiza*.** (A) In the phylogenetic tree of S. miltiorrhiza and Arabidopsis PYL members, the red-circled TRINITY_DN2396_c0_g1 is SmPYL8, with node reliability assessed by 1000-replicate bootstrap analysis. **(B)** Analysis of expression patterns of PYL members in *S. miltiorrhiza* following ABA treatment. 0H, 0.5H, 1H, 2H, 4H, 8H represent different time points following ABA treatment. The red circle marks SmPYL8.

**Fig. S8 ABA-induced expression pattern of *SmPYL8*.** Expression level of *SmPYL8* after ABA induction. *SmActin* served as the internal control. The values are means ± Standard Deviation (SD) of triplicate analyses. The significance levels were assessed using ANOVA, **P* < 0.05, ****P* < 0.001.

**Fig. S9 Vector construction and PCR detection for *SmPYL8***. (A) Overexpression (pCAMBIA2306) vector construction of *SmPYL8*. (B) PCR detection of *SmPYL8* overexpression lines. “M” represents DNA marker; “-” represents the negative control without DNA; “+” represents the positive control with added plasmid DNA. (C) PCR analysis of *SmPYL8*-RNAi lines. Lane M: DNA marker; Lane -: negative control (no template); Lane +: positive control (plasmid DNA). (D) The relative expression level of the *SmPYL8* gene in *SmPYL8* overexpression hairy roots. Error bars represent the ± Standard Deviation (SD) (ANOVA: ***P* < 0.01, ****P* < 0.001). (E) Expression analysis of *SmPYL8* in *SmPYL8*-RNAi hairy roots. The data are shown as mean ± SD (ANOVA; ***P* < 0.01).

**Fig. S10 SmMYB32 influenced the accumulation of phenolic acids and the expression of key enzyme genes involved in phenolic acid biosynthesis.** (A) Determination of phenolic acid content in *SmMYB32*-OE lines. TS represents total phenolic acids, which is the sum of RA and Sal B content. Error bars represent the ± Standard Deviation (SD) (ANOVA: **P* < 0.05, ***P* < 0.01, ****P* < 0.001). (B) Phenolic acid content in *SmMYB32*-RNAi lines. TS represents total phenolic acids, containing RA and Sal B. Error bars represent the ± Standard Deviation (SD) (ANOVA: **P* < 0.05, ***P* < 0.01, ****P* < 0.001). (C) Expression of phenolic acid biosynthesis genes in *SmMYB32*-OE lines. *SmActin* served as the internal reference gene. Error bars represent the ± standard deviation (SD), and significance is calculated by ANOVA (**P* < 0.05, ***P* < 0.01, ****P* < 0.001). (D) Detection of phenolic acid biosynthesis gene expression levels in *SmMYB32*-RNAi lines. *SmActin* was used as the internal reference gene. Error bars represent the ± standard deviation (SD), and significance is calculated by ANOVA (**P* < 0.05, ***P* < 0.01, ****P* < 0.001).

**Fig. S11 SmPYL8 influenced the accumulation of phenolic acids and the expression of key enzyme genes involved in phenolic acid biosynthesis.** (A) Determination of phenolic acid content in *SmPYL8* transgenic lines and control lines. TS represents total phenolic acids, which is the sum of RA and Sal B content. Error bars represent the standard deviation (ANOVA: ***P* < 0.01, ****P* < 0.001). (B) Detection of phenolic acid biosynthesis gene expression levels in *SmPYL8* transgenic line and control lines. *SmActin* served as the internal reference gene. Error bars represent the standard deviation, and significance is calculated by ANOVA (**P* < 0.05, ****P* < 0.001).

**Table S1** ABA-induced and tissue expression data for 26 candidate genes

| Gene ID | 0H | 0.5H | 1H | 2H | 4H | 8H | F | S | L | R |
| --- | --- | --- | --- | --- | --- | --- | --- | --- | --- | --- |
| TRINITY_DN352_c0_g1 | 120.19 | 644.16 | 677.52 | 96.56 | 158.36 | 182.62 | 235.134 | 360.784 | 0 | 1022.24 |
| TRINITY_DN9729_c0_g1 | 11.52 | 68.46 | 504.63 | 462.47 | 534.18 | 508.73 | 1.16142 | 3.72418 | 0.308383 | 17.1361 |
| TRINITY_DN25199_c0_g9 | 95.55 | 431.79 | 583.61 | 316.2 | 261.85 | 260.27 | 346.926 | 128.265 | 1.16752 | 427.308 |
| TRINITY_DN39845_c0_g1 | 2.14 | 70.62 | 242.32 | 137.67 | 63.03 | 69.6 | 2.92959 | 5.22967 | 0.433158 | 7.0191 |
| TRINITY_DN15648_c0_g1 | 44.44 | 518.57 | 318.07 | 204.79 | 173.68 | 115.92 | 190.152 | 205.184 | 6.36724 | 673.812 |
| TRINITY_DN13534_c0_g1 | 6.18 | 414.15 | 997.1 | 614.25 | 611.34 | 453.94 | 363.94 | 38.2695 | 0 | 80.1464 |
| TRINITY_DN24104_c1_g3 | 17.6 | 97.24 | 228.93 | 151.58 | 98.11 | 25.28 | 34.7007 | 22.8256 | 0.987632 | 48.4368 |
| TRINITY_DN24104_c1_g1 | 18.61 | 133.76 | 295.84 | 127.83 | 38.56 | 3.28 | 34.7007 | 22.8256 | 0.987632 | 48.4368 |
| TRINITY_DN15145_c0_g1 | 8.31 | 221.55 | 536.28 | 425.16 | 374.76 | 332.83 | 374.171 | 34.9145 | 5.31398 | 137.371 |
| TRINITY_DN23197_c0_g3 | 75.79 | 109.12 | 184.93 | 312.57 | 225.18 | 144.31 | 38.9556 | 94.0648 | 68.5822 | 196.142 |
| TRINITY_DN23941_c3_g2 | 88.96 | 158.26 | 398.13 | 320.68 | 198.1 | 128.7 | 56.836 | 0.692193 | 1.6985 | 7.8039 |
| TRINITY_DN23241_c1_g1 | 84.17 | 293.37 | 325.11 | 257.58 | 255.98 | 210.7 | 76.8356 | 77.4556 | 8.50392 | 159.971 |
| TRINITY_DN19352_c0_g1 | 35.25 | 155.85 | 209.25 | 37.13 | 26.34 | 24.69 | 12.787 | 28.8611 | 11.9637 | 48.1892 |
| TRINITY_DN17892_c0_g1 | 39.33 | 216.49 | 189.56 | 130.64 | 130.04 | 89.72 | 20.2791 | 23.8475 | 0.682709 | 52.5128 |
| TRINITY_DN20001_c0_g1 | 43.56 | 118.84 | 237.24 | 86.51 | 75.58 | 36.71 | 0.045511 | 0.944865 | 0.351983 | 0.316176 |
| TRINITY_DN17125_c1_g1 | 48.31 | 214.47 | 1188.8 | 1533.91 | 1355.62 | 1160.6 | 52.3463 | 48.3703 | 75.3607 | 134.023 |
| TRINITY_DN4096_c0_g1 | 1.38 | 45.84 | 278.17 | 309.18 | 285.99 | 245.58 | 24.5995 | 54.4205 | 98.6628 | 82.095 |
| TRINITY_DN17714_c0_g1 | 374.47 | 232.43 | 106.78 | 69.19 | 121.55 | 173.23 | 42.4326 | 53.6413 | 91.2235 | 75.2511 |
| TRINITY_DN13623_c0_g1 | 210.76 | 137.29 | 76.08 | 45.65 | 73.17 | 100.01 | 19.2044 | 159.583 | 48.534 | 253.818 |
| TRINITY_DN15349_c0_g1 | 20.96 | 95.63 | 268.98 | 207.13 | 100.11 | 82.52 | 53.5898 | 27.4886 | 124.544 | 91.4821 |
| TRINITY_DN22568_c0_g1 | 49.55 | 127.33 | 371.74 | 169.32 | 101.06 | 68.87 | 60.4839 | 30.1725 | 143.596 | 58.3919 |
| TRINITY_DN18990_c1_g1 | 24.33 | 75.7 | 248.73 | 163.64 | 182.19 | 170.17 | 14.8472 | 14.3113 | 15.6441 | 25.8233 |
| TRINITY_DN22905_c0_g1 | 6.24 | 87.81 | 250.63 | 112.92 | 84.97 | 73.21 | 15.1746 | 17.0906 | 3.10288 | 38.9608 |
| TRINITY_DN6735_c0_g1 | 48.23 | 178.9 | 223.34 | 138.91 | 104.29 | 96.67 | 32.1359 | 34.7631 | 2.49384 | 125.453 |
| TRINITY_DN12190_c0_g1 | 13.55 | 43.45 | 226.01 | 273.61 | 113.32 | 91.09 | 1.54627 | 3.41788 | 3.99942 | 1.65586 |
| TRINITY_DN39938_c0_g1 | 281.18 | 251.53 | 214.92 | 134.46 | 124.47 | 55.28 | 1.17954 | 0 | 0 | 0.818392 |

**Table S2** ABA-induced expression data of 11 PYL genes in *S. miltiorrhiza*

| Gene ID | 0H | 0.5H | 1H | 2H | 4H | 8H |
| --- | --- | --- | --- | --- | --- | --- |
| TRINITY_DN5107_c0_g2 | 143.46 | 113.68 | 87.4 | 45.38 | 48.96 | 35.38 |
| TRINITY_DN2396_c0_g1 | 96.36 | 90.56 | 82.39 | 43.35 | 66.36 | 81.76 |
| TRINITY_DN2605_c2_g1 | 3.33 | 1.72 | 1.1 | 0.22 | 3.91 | 0 |
| TRINITY_DN11812_c0_g2 | 162.77 | 150.72 | 98.5 | 87.39 | 92.58 | 78.78 |
| TRINITY_DN2605_c0_g2 | 14.9 | 12.31 | 7.01 | 8.8 | 8.68 | 2.77 |
| TRINITY_DN13627_c0_g1 | 50.37 | 33.89 | 23.91 | 17.34 | 27.63 | 20.88 |
| TRINITY_DN2605_c1_g1 | 160.03 | 36.82 | 7.43 | 6.1 | 6.82 | 3.55 |
| TRINITY_DN33124_c0_g1 | 0 | 0 | 0 | 0 | 0 | 0 |
| TRINITY_DN2605_c0_g1 | 0 | 0 | 0 | 0 | 0 | 0 |
| TRINITY_DN2605_c1_g1 | 59.27 | 26.98 | 5.07 | 9.21 | 7.79 | 5.52 |
| TRINITY_DN5138_c0_g3 | 2.55 | 2.37 | 3.84 | 6.11 | 2.57 | 2.45 |

**Table S3** Primer names and sequences used in this study

| Primers | Primer sequences (5’-3’) | Purpose |
| --- | --- | --- |
| pGEX4T-1-SmMYB32-F | CGGATCCCCGGAATTCATGGATCGGATCAAAGGTCCATG | Pull down |
| pGEX4T-1-SmMYB32-R | ATGCGGCCGCTCGAGATCAACCTTGCTCATCCCCATCTG | Pull down |
| pCold-SmPYL8-F | CTCGGTACCCTCGAGGGATCCATGATGAACATCAAAGGAT | Pull down |
| pCold-SmPYL8-R | CTATCTAGACTGCAGGTCGACCGCACGGTTGATGGGTTCA | Pull down |
| pXY104-SmPYL8-F | TACAATTACAGGTACCCGGGATGATGAACATCAAAGGAT | BiFC |
| pXY104-SmPYL8-R | CACCGCCGTCGACTCTAGACGCACGGTTGATGGGTTCA | BiFC |
| pXY106-SmMYB32-F | GCTCTAGAATGGATCGGATCAAAGGTCCATG | BiFC |
| pXY106-SmMYB32-R | GCTCTAGAATCAACCTTGCTCATCCCCATCTG | BiFC |
| pHB-SmMYB32-F | ACCAGTCTCTCTCTCAAGCTTATGGATCGGATCAAAGGTCCATG | Plant overexpression, Subcellular localization |
| pHB-SmMYB32-R | TGAGCTCCTGCAGCTCGAGGATCCATCAACCTTGCTCATCCCCATCTG | Plant overexpression, Subcellular localization |
| pFGC5941-SmMYB32-F1 | TTACATTTACAATTACCATGGTCCACCACCCGCTCAAG | RNAi expression vector |
| pFGC5941-SmMYB32-R1 | TTAAATCATCGATTGGGCGCGCCACGATGTGCGGCTGCG | RNAi expression vector |
| pFGC5941-SmMYB32-F2 | GGTCTTAATTAACTCTCTAGAGGAGGTCCACCACCCGCTCAAGC | RNAi expression vector |
| pFGC5941-SmMYB32-R2 | AATTTGCAGGTATTTGGATCCGCGGGCGACGGGGCGGTAG | RNAi expression vector |
| pCAMBIA2306-SmPYL8-F | ACGAGCTCGGTACCCGGGGATCCATGATGAACATCAAAGGATTGAGCGCG | Plant overexpression |
| pCAMBIA2306-SmPYL8-R | AGGGCGAATTGGTCGACTCTAGACGCACGGTTGATGGGTTCAGTC | Plant overexpression |
| pFGC5941-SmPYL8-F1 | TTACATTTACAATTACCATGGATCAACCACAGAAGTACAAACCTTT | RNAi expression vector |
| pFGC5941-SmPYL8-R1 | TTAAATCATCGATTGGGCGCGCCTCATTATCATCAAGAAGCTCCAATC | RNAi expression vector |
| pFGC5941-SmPYL8-F2 | GGTCTTAATTAACTCTCTAGAATCAACCACAGAAGTACAAACCTTT | RNAi expression vector |
| pFGC5941-SmPYL8-R2 | AATTTGCAGGTATTTGGATCCTCATTATCATCAAGAAGCTCCAATC | RNAi expression vector |
| rolB-F | GCTCTTGCAGTGCTAGATTT | PCR identification |
| rolB-R | GAAGGTGCAAGCTACCTCTC | PCR identification |
| pGADT7-SmPYL8-F | CCATGGAGGCCAGTGAATTCATGATGAACATCAAAGGATTGAGCGCG | Yeast two hybrid |
| pGADT7-SmPYL8-R | GCTCGAGCTCGATGGATCCCGCACGGTTGATGGGTTCAGTC | Yeast two hybrid |
| pGBKT7-SmMYB32-F | CATATGGCCATGGAGGCCGAATTCATGGATCGGATCAAAGGTCCATG | Yeast two hybrid |
| pGBKT7-SmMYB32-R | CGGCCGCTGCAGGTCGACGGATCCATCAACCTTGCTCATCCCCATCTG | Yeast two hybrid |
| pAbAi-proSmCYP76AH3-MBS1-F | AGCTATTCCAGTTAAATAATTCCAGTTAAATAATTCCAGTTAAATA | Yeast one hybrid |
| pAbAi-proSmCYP76AH3-MBS1-R | TCGATATTTAACTGGAATTATTTAACTGGAATTATTTAACTGGAAT | Yeast one hybrid |
| pAbAi-proSmCYP71D411-MBS1-F | AGCTGTAGTGGTTGAGTTGTAGTGGTTGAGTTGTAGTGGTTGAGTT | Yeast one hybrid |
| pAbAi-proSmCYP71D411-MBS1-R | TCGAAACTCAACCACTACAACTCAACCACTACAACTCAACCACTAC | Yeast one hybrid |
| EMSA-SmCYP76AH3pro-MBS1-F | ATTCCAGTTAAATAATTCCAGTTAAATAATTCCAGTTAAATA | EMSA |
| EMSA-SmCYP76AH3pro-MBS1-R | TATTTAACTGGAATTATTTAACTGGAATTATTTAACTGGAAT | EMSA |
| EMSA-SmCYP71D411pro-MBS1-F | GTAGTGGTTGAGTTGTAGTGGTTGAGTTGTAGTGGTTGAGTT | EMSA |
| EMSA-SmCYP71D411pro-MBS1-R | AACTCAACCACTACAACTCAACCACTACAACTCAACCACTAC | EMSA |
| EMSA-Mutant-SmCYP76AH3pro-MBS1-F | ATTCAAAAAAAATAATTCAAAAAAAATAATTCAAAAAAAATA | EMSA |
| EMSA-Mutant-SmCYP76AH3pro-MBS1-R | TATTAAAAAAGAATTATTAAAAAAGAATTATTAAAAAAGAAT | EMSA |
| EMSA-Mutant-SmCYP71D411pro-MBS1-F | GTAGAAAAAAAGTTGTAGAAAAAAAGTTGTAGAAAAAAAGTT | EMSA |
| EMSA-Mutant-SmCYP71D411pro-MBS1-R | AACTAAAAAACTACAACTAAAAAACTACAACTAAAAAACTAC | EMSA |
| qRT-PCR-SmCYP76AH1-F | TGCCCAACTTCGCCGACTACTTC | Real-time PCR |
| qRT-PCR-SmCYP76AH1-R | GTGTTCGTGTCCGATCCTCCCAC | Real-time PCR |
| qRT-PCR-SmCPS-F | GATCGCCTCGTCAATACCAT | Real-time PCR |
| qRT-PCR-SmCPS-R | TTCGAACCCACAAGTCATGT | Real-time PCR |
| qRT-PCR-SmKSL-F | GTGTGACCCTTCTGCTAGCA | Real-time PCR |
| qRT-PCR-SmKSL-R | TGCATTGTCTTGGGAAGATG | Real-time PCR |
| qRT-PCR-SmGGPPS-F | GCTGTGCTCGCAGGGGATG | Real-time PCR |
| qRT-PCR-SmGGPPS-R | ATCGCCGGTGCAGTTCAGG | Real-time PCR |
| qRT-PCR-SmDXR-F | CGACGAGAAAATCGGATACCTGG | Real-time PCR |
| qRT-PCR-SmDXR-R | CATACAAGAGCAGGACTCGAACCG | Real-time PCR |
| qRT-PCR-SmCYP76AH3-F | CAAGTCGTCAACGGCTACCTCATC | Real-time PCR |
| qRT-PCR-SmCYP76AH3-R | GGTTGGAAGGCGTCTGGATTGTTC | Real-time PCR |
| qRT-PCR-SmCYP71D373-F | ACTGCCTAGAATGAGTTCCCAAAGATG | Real-time PCR |
| qRT-PCR-SmCYP71D373-R | ATGCGTTCACTATCACTCTCGTTCC | Real-time PCR |
| qRT-PCR-SmHMGR-F | TCGTTTTCAATAAGTCGAGTAGA | Real-time PCR |
| qRT-PCR-SmHMGR-R | ATTCTGAAGGAAGTCCAAAACAT | Real-time PCR |
| qRT-PCR-SmCYP76AK1-F | CAAGCACGAGACTCCTCCGATTAC | Real-time PCR |
| qRT-PCR-SmCYP76AK1-R | GGTGTCCGATCCTGCGATGATTATG | Real-time PCR |
| qRT-PCR-SmCYP71D375-F | AGTGAATGCATGGGCACTGG | Real-time PCR |
| qRT-PCR-SmCYP71D375-R | AGCATAGCAAGCGGAAGCTC | Real-time PCR |
| qRT-PCR-SmCYP71D411-F | AGTTCATTCTTGCGACGCTTCTCTATC | Real-time PCR |
| qRT-PCR-SmCYP71D411-R | ATGCCTCCACCACATCCAATTCATC | Real-time PCR |
| qRT-PCR-SmTAT-F | CAACTGCTGGTCTTCCACAAAC | Real-time PCR |
| qRT-PCR-SmTAT-R | GCGAGCCAAAACGGACA | Real-time PCR |
| qRT-PCR-SmPAL-F | GATAGCGGAGTGCAGGTCGTAC | Real-time PCR |
| qRT-PCR-SmPAL-R | CGAACTAGCAGATTGGCAGAGG | Real-time PCR |
| qRT-PCR-SmC4H-F | CCAGGAGTCCAAATAACAGAGC | Real-time PCR |
| qRT-PCR-SmC4H-R | GCCACCAAGCGTTCACCAAGAT | Real-time PCR |
| qRT-PCR-Sm4CL-F | CAGCAAGTGGACGGCGAGAATC | Real-time PCR |
| qRT-PCR-Sm4CL-R | GACACGCAGACAGCAGAGCATC | Real-time PCR |
| qRT-PCR-SmHPPR-F | TGACTCCAGAAACAACCCACATT | Real-time PCR |
| qRT-PCR-SmHPPR-R | CCCAGACGACCCTCCACAAG | Real-time PCR |
| qRT-PCR-SmRAS-F | CGAGATCGCCTACTCCAAGTTCAAG | Real-time PCR |
| qRT-PCR-SmRAS-R | AGATGGCGTTACCGAAGTACCCC | Real-time PCR |
| qRT-PCR-SmCYP98A14-F | GGTCTGTACCGTCGTCCTCTTCTCC | Real-time PCR |
| qRT-PCR-SmCYP98A14-R | CCTTTTCCCAAATACCAGCCTTGT | Real-time PCR |
| qRT-PCR-SmMYB32-F | GCCGAGAAACTGGTCCCTGATTAG | Real-time PCR |
| qRT-PCR-SmMYB32-R | GCCTTGATGATGGTGTCGTCCTC | Real-time PCR |
| qRT-PCR-SmPYL8-F | TGGATGTGCCAGAAGGGAAC | Real-time PCR |
| qRT-PCR-SmPYL8-R | ACGGTTGATGGGTTCAGTCC | Real-time PCR |
| qRT-PCR-SmActin-F | AGCACCGAGCAGCATGAAGATT | Real-time PCR |
| qRT-PCR-SmActin-R | AGCAAAGCAGCGAACGAAGAGT | Real-time PCR |

**>SmMYB32 CDS (GenBank: KF059386.1)**

ATGGATCGGATCAAAGGTCCATGGAGCCCCGAGGAGGACGATTTGCTGCAGAGACTGGTGGAGAAGCACGGGCCGAGAAACTGGTCCCTGATTAGCAAATCAATCCCGGGGAGATCGGGCAAATCGTGCCGGCTGCGGTGGTGCAACCAGCTGTCGCCGCAAGTGGAGCATCGGGCCTTCACGGCGGAGGAGGACGACACCATCATCAAGGCCCACGCCAAGTTCGGGAACAAGTGGGCCACCATAGCCCGATTCCTCTCGGGCCGGACCGACAACGCCATCAAAAACCACTGGAACTCCACGCTCAAGCGCAAGTGCTTCTCCATGTCGGAGGAGCTCACCGACTTCGACCCGGAGGTCCACCACCCGCTCAAGCGATCCGCCAGTGCCGGCCCGCCCGGCTCGGCCTTCTGCTTCAACCCGGGCAGCCCCTCCGGGTCCGACGATTCGGGCCACTCGGCCCACCCCGTCTACCGCCCCGTCGCCCGCCCCGCTCCGCAGCCGCACATCGTCACATCGCTCAGCCTCTCCCTGCCCGGAACCGAACCCGCACCCGCCGTGATGGATCCGGATCCCGATCCTCAGCCGCGATGGGCGGCGGAGAAGCAGTTTTTCAGCGCGGAATTTCTGGGGGTGATGCAGGAGATTATAAGGAAGGAGGTGAGGAATTACATGGTGGGGATGGAGATGGAGCACAAGGGGCAGTGCATGCAGAGTGATGCGATGATGATTAATGGCGTAATCAAGCAGATGGGGATGAGCAAGGTTGATTGA

**>SmPYL8 CDS (GenBank: XM_057942659.1)**

ATGATGAACATCAAAGGATTGAGCGCGGTGGAGAGTGAGTATACACTGAAGCATCACAGGCACCAGATTAAGGATAATCAATGCAGTTCGTTCCTCATCAAGCATATTAAAGCGCCCGTACATCTTGTTTGGTCTCTGGTCAGGAGGTTTGATCAACCACAGAAGTACAAACCTTTTGTTAGCCGTTGCATTGTGCAGGGAAATCTTGAAATTGGCAGTCTGAGGGAAGTTGATGTCAAGTCGGGTCTCCCTGCTACAACCAGCACCGAGAGATTGGAGCTTCTTGATGATAATGAACACATACTTAGCGTAAGGATCGTTGGTGGAGACCACAGACTCAGAAACTACTCTTCCATGGTGTCTGTTCATCCTGAGGTCATTGACGGGAGACCTGGGACAGTGGTGATTGAATCGTTTGTGGTGGATGTGCCAGAAGGGAACACAAAAGACGAAACATGCTACTTTGTCGAAGCACTGATCAGGTGTAATCTGAAATCACTGGCTGATGTTTCGGAGAGGCTGGCTGTGCAAGACAGGACTGAACCCATCAACCGTGCGTAA
